# Presence of ´isolated´ tricuspid regurgitation should prompt the suspicion of heart failure with preserved ejection fraction

**DOI:** 10.1371/journal.pone.0171542

**Published:** 2017-02-15

**Authors:** Julia Mascherbauer, Andreas A. Kammerlander, Caroline Zotter-Tufaro, Stefan Aschauer, Franz Duca, Daniel Dalos, Susanne Winkler, Matthias Schneider, Jutta Bergler-Klein, Diana Bonderman

**Affiliations:** From the Department of Cardiology, Medical University of Vienna, Vienna General Hospital, Vienna, Austria; Scuola Superiore Sant'Anna, ITALY

## Abstract

**Background:**

Diastolic dysfunction of the left ventricle is common but frequently under-diagnosed. Particularly in advanced stages affected patients may present with significant functional tricuspid regurgitation (TR) as the most prominent sign on echocardiography. The underlying left ventricular pathology may eventually be missed and symptoms of heart failure are attributed to TR, with respective therapeutic consequences.

The aim of the present study was to determine prevalence and mechanisms underlying TR evolution in heart failure with preserved ejection fraction (HFpEF).

**Methods and results:**

Consecutive HFpEF patients were enrolled in this prospective, observational study. Confirmatory diagnostic tests including echocardiography and invasive hemodynamic assessments were performed.

Of the 175 patients registered between 2010 and 2014, 51% had significant (moderate or severe) TR without structural abnormalities of the tricuspid valve. Significant hemodynamic differences between patients with and without relevant TR were encountered. These included elevated pulmonary vascular resistance (p = 0.038), reduced pulmonary arterial compliance (PAC, p = 0.005), and elevated left ventricular filling pressures (p = 0.039) in the TR group. Multivariable binary logistic regression analysis revealed diastolic pulmonary artery pressure (p = 0.029) and PAC (p = 0.048) as independent determinants of TR.

Patients were followed for 18.1±14.1 months, during which 32% had a cardiac event. While TR was associated with outcome in the univariable analysis, it failed to predict event-free survival in the multivariable model.

**Conclusions:**

The presence of ´isolated´ functional TR should prompt the suspicion of HFpEF. Our data show that significant TR is a marker of advanced HFpEF but neither an isolated entity nor independently associated with event-free survival.

## 1. Introduction

Diastolic dysfunction of the left ventricle that may convert into the clinical picture of heart failure with preserved ejection fraction (HFpEF) is a growing health care problem [1, 2]. We have observed that in advanced disease stages affected patients frequently present with significant functional tricuspid regurgitation (TR) as the most prominent sign on echocardiographic examination. The underlying left ventricular pathology may eventually be missed and symptoms of heart failure are attributed to TR. Although current guidelines on the management of valvular heart disease lack any recommendation for the treatment of ´isolated´ functional TR [3, 4] affected patients may be referred to surgical repair [5]. In the present work we hypothesized that hemodynamic alterations characteristic of HFpEF [6–8] cause functional TR.

## 2. Methods

### 2.1 Study design

The present study was undertaken in order to determine the prevalence of significant TR in HFpEF patients, the underlying mechanisms, and its impact on event-free survival. This was a prospective observational study performed at the Medical University of Vienna, approved by the local ethics committee (Ethics Committee of the Medical University of Vienna, EK #796/2010). All participants gave written informed consent.

After thorough baseline evaluation, patients were followed by ambulatory visits and phone calls at 6-month intervals. A combined endpoint consisting of hospitalization for heart failure or death from cardiac causes was defined as the main outcome measure. Endpoints were adjudicated by our adjudication committee consisting of CZT and AAK, who were blinded to hemodynamics and other patient characteristics. An age-matched control group was also assessed with respect to clinical characteristics and imaging parameters.

### 2.2 Diagnosis of heart failure with preserved ejection fraction

Consecutive patients with HFpEF were enrolled. HFpEF was diagnosed in the presence of: (1) symptoms or signs of heart failure; (2) normal or mildly reduced LV systolic function (LV ejection fraction (EF) >50%); and (3) evidence of abnormal LV relaxation or diastolic stiffness [1, 2].

Reasons for exclusion were: significant coronary artery disease as diagnosed by coronary angiography, significant aortic or mitral valve disease, congenital heart disease, and cardiac amyloidosis as diagnosed by cardiac magnetic resonance imaging (CMR) and / or endomyocardial biopsy. Furthermore, patients with chronic lung disease such as bronchial asthma or chronic obstructive pulmonary disease (COPD) with FEV_1_ <60% of predicted, restrictive lung disease with TLC <60% of predicted and / or current O_2_ therapy were excluded from the study.

The hemodynamic diagnosis of HFpEF was confirmed, if the pulmonary artery wedge pressure (PAWP) exceeded 12 mm Hg [1].

### 2.3 Assessment of tricuspid regurgitation

All echo studies were performed by board certified physicians, using high-end scanners such as GE Vivid 5 and Vivid 7 (GE Healthcare, Wauwatosa, WI, USA). The evaluation included M-mode and 2-dimensional echocardiography, as well as conventional and color Doppler ultrasonography according to current recommendations [9–11].

TR was quantified by an integrated approach (Table 1) [9, 11, 12]. Moderate and severe TR were considered significant and were compared with trace and mild TR. The graduation into non-significant and significant TR was chosen to account for inaccuracies due to the semiquantitative assessment of TR by echocardiography and has previously been deemed reasonable [5, 13, 14].

**Table 1:** Echocardiographic parameters used for grading of tricuspid regurgitation severity [9].

Parameter	Mild	Moderate	Severe
Tricuspid valve	Normal	Normal or abnormal	Abnormal / flail leaflet / poor coaptation
RV/RA/IVC size	Normal	Normal or dilated	Dilated
VC width [mm]	Not defined	Not defined, but <7	>7
PISA radius [mm]	≤5	6–9	>9
Hepatic vein flow	Systolic dominance	Systolic blunting	Systolic reversal

RV indicates right ventricle; RA, right atrium; IVC, inferior vena cava; VC, vena contracta; PISA, proximal isovelocity surface area.

LVEF was assessed with the biplane Simpson´s method. Right ventricular (RV) function was assessed by the percentage RV fractional area change (FAC), defined as (end-diastolic area—end-systolic area) / end-diastolic area x 100, according to recent recommendations [15]. In addition, tricuspid annular plane systolic excursion (TAPSE) was measured [15]. RV dysfunction was defined as FAC < 35% and TAPSE <16 mm [15].

### 2.4 Influence of TR on imaging and hemodynamic parameters

Imaging and hemodynamic variables were split into two groups: 1. influenced by TR (i.e. TR- dependent) and 2. not influenced by TR (TR- independent). Changes in right heart segments and pressures were considered as TR- dependent, while pulmonary vasculature and left heart segments were defined as TR- independent.

### 2.5 Right and left heart catheterization

For right heart catheterization a 7F Swan-Ganz catheter (Baxter, Irvine, CA) was inserted via a jugular or femoral access. Filling pressures were averaged after recording of eight heart cycles using CathCorLX (Siemens AG, Berlin and Munich, Germany). PAWP, pulmonary arterial pressure (PAP), and cardiac output (CO), were determined. CO was measured by both thermodilution and Fick method. Simultaneously, all patients underwent direct assessment of LV filling pressures, followed by coronary angiography. Standard formulae were used for the calculation of hemodynamic parameters[16, 17].

### 2.6 Cardiac magnetic resonance imaging

CMR was primarily used as a complimentary method for the assessment of RV function. All patients without pacemaker or other precluding conditions underwent CMR at baseline, using a 1.5-T scanner (Avanto, Siemens Medical Solutions, Erlangen, Germany). Functional and late gadolinium enhancement imaging studies were performed according to standard protocols [18]. RV dysfunction was defined as RVEF <45%. Two independent observers (SA, AB) blinded to clinical data read all CMR studies.

### 2.7 Statistical analysis

Categorical variables were reported in percent and/or total numbers. Continuous data are presented as mean ± standard deviation. Baseline characteristics were compared using chi square or Fisher´s exact test for categorical variables and Wilcoxon two sample test for continuous variables. Variables were stratified into TR-dependent (right atrial (RA) and RV size, RV function, RA pressure, and PAP) and TR-independent (all others; see Tables 2–4). Binary logistic regression analysis was used to identify TR-independent parameters associated with the presence of significant TR. A multivariable regression model using a stepwise approach was run for clinical, hemodynamic and imaging parameters, respectively. To identify variables associated with cardiac events, a univariable Cox regression model was performed for each influence factor, followed by a multiple Cox regression model with stepwise backward selection. This was done for clinical, hemodynamic and imaging parameters.

**Table 2:** Baseline clinical characteristics of HFpEF patients and controls.

	HFpEF patients (n = 175)	Controls (n = 45)	p-value
Baseline Characteristics			
Age (years)	71.0±8.7	69.9±7.2	0.263
Female (%)	69.1	53.3	**0.046**
BMI (kg/m^2^)	30.9±7.0	28.4±4.7	**0.018**
Atrial Fibrillation (%)	60.6	8.0	**0.001**
Diabetes (%)	38.6	18.3	**0.008**
Arterial Hypertension (%)	97.7	91.0	0.070
CAD (%)	20.5	N/A	N/A
COPD, mild (%)	37.8	24.6	0.096
History of smoking (%)	34.3	35.6	0.895
Pacemaker (%)	10.5	0.0	**0.024**
NYHA (%)			**<0.001**
NYHA II	30.7	0.0	
NYHA III-IV	69.3	0.0	
SBP (mmHg)	137.4±21.0	150.9±11.4	**<0.001**
DBP (mmHg)	78.2±12.9	69.4±11.3	**<0.001**
Heart rate (bpm)	72.3±14.8	65.7±11.5	**0.008**
NT-proBNP (pg/ml)	1876.6±2916.5	288.9±227±3	**<0.001**
GFR (ml/1.73m^2^)	59.1±20.0	68.4±11.8	**0.001**

BMI indicates body mass index; CAD, coronary artery disease as assessed by coronary angiography; COPD, chronic obstructive pulmonary disease; NYHA, New York Heart Association functional class; SBP, systolic blood pressure; DBP, diastolic blood pressure; GFR, glomerular filtration rate.

**Table 3:** Baseline imaging characteristics of HFpEF patients and controls.

	HFpEF patients (n = 175)	Controls (n = 45)	p-value
**Echocardiography**			
**TR- dependent**			
RA diameter (mm)	63.0±9.6	51.5±5.1	**<0.001**
RA area (cm^2^)	26.8±9.3	18.5±4.2	**<0.001**
RVEDD (mm)	37.4±7.6	31.8±3.6	**<0.001**
TAPSE (mm)	19.6±5.8	24.6±3.1	**<0.001**
sPAP (mmHg)	60.0±17.8	38.4±8.4	**<0.001**
Significant TR (%)	51.2	0.0	**<0.001**
**TR- independent**			
LA diameter (mm)	64.2±8.3	52.5±5.8	**<0.001**
LA area (cm^2^)	29.3±7.1	22.7±4.1	**<0.001**
LVEDD (mm)	45.1±5.8	44.0±3.5	0.326
LVEF (%)	60.4±9.4	59.6±8.9	0.164
IVS (mm)	12.6±2.0	12.6±1.5	0.586
E/E’	16.2±7.4	9.5±3.7	**<0.001**
E/A	2.4±2.6	0.9±1.6	**<0.001**
**Cardiac magnetic resonance imaging**			
**TR- dependent**			
RA diameter (mm)	65.1±9.2	56.0±6.6	**<0.001**
RA area (cm^2^)	29.1±10.2	22.2±3.7	**<0.001**
RVEDD (mm)	39.6±7.5	36.5±3.9	**0.008**
RVEDV (ml)	157.7±111.6	126.8±29.8	**0.048**
RVEF (%)	52.7±11.0	57.6±7.4	**0.007**
**TR- independent**			
LA diameter (mm)	65.2±9.2	56.3±6.4	**<0.001**
LA area (cm^2^)	31.2±9.4	25.0±5.1	**<0.001**
LVEDD (mm)	47.6±5.9	47.1±6.7	0.533
IVS (mm)	11.4±2.2	11.1±1.5	0.580
LVEDV (ml)	127.5±46.2	127.2±24.9	0.422
LVEF (%)	63.3±11.2	68.5±6.6	0.004
CO (l/min)	5.3±1.8	5.7±1.4	0.105

Parameters are listed according to right heart segments (TR-dependent) versus right heart afterload (TR-independent). Changes in right heart segments are considered consequences of both right heart afterload as well as TR, while parameters of right ventricular afterload are not thought to be influenced by TR.

TR indicates tricuspid regurgitation; RA, right atrium; RVEDD, right ventricular end-diastolic diameter; RV FAC, right ventricular fractional area change; TAPSE, tricuspid annular plane systolic excursion; sPAP, systolic pulmonary artery pressure; LA, left atrium; LVEDD, left ventricular end-diastolic diameter; LVEF, left ventricular ejection fraction; IVS, interventricular septal thickness; E/A, ratio of early to late ventricular filling velocities; E/E’, ratio of transmitral early peak velocity to septal mitral annulus velocity; RVEDV, right ventricular end-diastolic volume; RVEF, right ventricular ejection fraction; LVEDV, left ventricular end-diastolic volume; CO, cardiac output.

**Table 4:** Baseline clinical characteristics of HFpEF patients, stratified by tricuspid regurgitation severity.

	All patients (n = 175)	Non-significant TR (48.8%)	Significant TR (51.2%)	p-value
Baseline Characteristics				
Age (years)	71.0±8.7	69.5±9.2	72.5±7.8	**0.026**
Female (%)	69.2	70.2	68.2	0.770
BMI (kg/m^2^)	30.9±7.0	31.4±6.6	29.8±6.3	0.172
Atrial Fibrillation (%)	60.6	41.5	78.4	**<0.001**
Diabetes (%)	38.6	38.6	38.6	0.991
Arterial Hypertension (%)	97.7	97.6	97.7	0.953
CAD (%)	20.5	24.1	17.0	0.253
COPD, mild (%)	37.8	41.2	34.7	0.423
History of smoking* (%)	34.3	36.1	32.6	0.623
Pacemaker (%)	10.5	9.6	11.4	0.713
NYHA (%)				**0.028**
NYHA II	30.7	38.8	22.9	
NYHA III-IV	69.3	61.3	77.2	
6-MWD (m)	319.9±123.0	341.6±114.1	300.0±128.4	**0.036**
SBP (mmHg)	137.4±21.0	141.9±20.5	133.7±20.7	**0.012**
DBP (mmHg)	78.2±12.9	79.5±12.0	77.4±13.6	0.249
Heart rate (bpm)	72.3±14.8	71.1±14.7	73.0±14.5	0.348
NT-proBNP (pg/ml)	1876.6±2916.5	1290.8±1901.1	2405.3±3545.1	**<0.001**
GFR (ml/1.73m^2^)	59.1±20.0	64.0±21.1	54.7±18.1	**0.008**
Beta-blocker (%)	73.3	71.0	75.0	0.586
Calcium channel blocker (%)	27.4	37.1	20.2	**0.024**
ARB (%)	35.6	43.5	29.8	0.086
ACE-I (%)	32.9	27.4	36.9	0.228
Diuretics (%)	76.0	67.7	82.1	**0.044**

TR indicates tricuspid regurgitation; BMI, body mass index; CAD, coronary artery disease; COPD, chronic obstructive pulmonary disease; NYHA, New York Heart Association functional class; 6-MWD, six-minute walk distance; SBP, systolic blood pressure; DBP, diastolic blood pressure; GFR, glomerular filtration rate; ARB, angiotensin II receptor blocker; ACE-I, angiotensin-converting-enzyme inhibitor.

* Only two patients were current smokers.

Statistical analyses were performed using SPSS Statistics Version 18 (IBM, Armonk, NY) and STATA version 12 (Stata Corp, College Station, TX). Statistical significance was set at p<0.05 for all tests. P-values were considered exploratory.

## 3. Results

### 3.1 Baseline characteristics

Between January 2010 and November 2014, 175 consecutive patients with a confirmed diagnosis of HFpEF and 45 age-matched control subjects were registered. Clinical and imaging characteristics of patients and controls are summarized in Tables 2 and 3. While 51% of HFpEF patients presented with significant TR, none of the control subjects had significant TR.

Baseline characteristics of HFpEF patients, stratified by tricuspid regurgitation severity, are displayed in Table 4.

In brief, patients with relevant TR were older (p = 0.026), more frequently presented with atrial fibrillation (p<0.001), and renal dysfunction (p = 0.008). Furthermore, TR patients were more symptomatic as measured by NYHA functional class (p = 0.028), had lower systolic blood pressures (p = 0.012), and shorter 6-minute walk distances (p = 0.036).

Table 5 lists imaging parameters with respect to the presence or absence of significant TR. Right heart dimensions were enlarged in TR patients (mean RA area, p<0.001; mean RV end-diastolic diameter, p<0.001), and RV systolic function by FAC was worse (p = 0.001) than in the comparator.

**Table 5:** Baseline imaging characteristics in HFpEF patients, stratified by tricuspid regurgitation severity.

	All (n = 175)	Non-significant TR (48.8%)	Significant TR (51.2%)	p-value
**Echocardiography**				
**TR- dependent**				
RA diameter (mm)	63.0±9.6	59.4±7.9	66.7±10.0	**<0.001**
RA area (cm^2^)	26.8±9.3	23.2±6.8	30.5±10.1	**<0.001**
RVEDD (mm)	37.4±7.6	34.5±6.3	40.4±7.6	**<0.001**
RV FAC (%)	41.0±12.8	44.4±12.5	37.6±12.3	**0.001**
TAPSE (mm)	19.6±5.8	20.9±5.2	18.2±6.1	**0.001**
sPAP (mmHg)	60.0±17.8	52.4±15.7	65.8±17.2	**<0.001**
**TR- independent**				
LA diameter (mm)	64.2±8.3	62.3±8.2	66.1±9.1	**0.003**
LA area (cm^2^)	29.3±7.1	28.0±6.9	30.7±7.0	**0.020**
LVEDD (mm)	45.1±5.8	45.2±6.0	45.0±5.6	0.790
LVEF (%)	60.4±9.4	61.4±10.5	59.2±8.2	0.263
IVS (mm)	12.6±2.0	12.9±2.0	12.4±1.9	**0.027**
E/E’	16.2±7.4	16.4±7.3	15.9±7.5	0.636
E/A	2.4±2.6	2.0±3.0	3.0±2.0	**<0.001**
**Cardiac magnetic resonance imaging (n = 122, 70% of all patients)**				
**TR- dependent**				
RA diameter (mm)	65.1±9.2	61.4±7.9	69.1±9.0	**<0.001**
RA area (cm^2^)	29.1±10.2	24.9±6.9	33.9±11.2	**<0.001**
RVEDD (mm)	39.6±7.5	36.9±6.2	42.8±7.7	**<0.001**
RVEDV (ml)	157.7±111.6	131.9±39.2	187.6±153.6	**0.001**
RVEF (%)	52.7±11.0	55.2±11.5	49.5±9.6	**0.006**
**TR-independent**				
LA diameter (mm)	65.2±9.2	62.6±8.3	68.0±9.6	**0.002**
LA area (cm^2^)	31.2±9.4	28.3±7.9	34.5±10.1	**<0.001**
LVEDD (mm)	47.6±5.9	47.3±6.2	47.4±5.4	0.238
IVS (mm)	11.4±2.2	11.9±2.3	10.9±2.0	**0.024**
LVEDV (ml)	127.5±46.2	130.1±54.4	124.4±35.7	0.923
LVEF (%)	63.3±11.2	64.3±11.8	62.3±10.7	0.223
CO (l/min)	5.3±1.8	5.4±2.1	5.2±1.6	0.985

Parameters are listed according to right heart segments versus right heart afterload. Changes in right heart segments are considered consequences of both right heart afterload as well as TR, while parameters of right ventricular afterload are not thought to be influenced by TR.

TR indicates tricuspid regurgitation; RA, right atrium; RVEDD, right ventricular end-diastolic diameter; RV FAC, right ventricular fractional area change; TAPSE, tricuspid annular plane systolic excursion; sPAP, systolic pulmonary artery pressure; LA, left atrium; LVEDD, left ventricular end-diastolic diameter; LVEF, left ventricular ejection fraction; IVS, interventricular septal thickness; E/A, ratio of early to late ventricular filling velocities; E/E’, ratio of transmitral early peak velocity to septal mitral annulus velocity; RVEDV, right ventricular end-diastolic volume; RVEF, right ventricular ejection fraction; LVEDV, left ventricular end-diastolic volume; CO, cardiac output.

Left atrial (LA) chamber dimensions were also enlarged in TR patients (p = 0.003) reflecting elevated LV filling pressures. Further significant differences were found with respect to interventricular septum thickness (p = 0.027) and E/A ratio (p<0.001).

Table 6 shows hemodynamic parameters with respect to the presence or absence of significant TR. Importantly, subtle but significant between-group differences were found with respect to invasively measured hemodynamic parameters of RV afterload, i.e. PVR (p = 0.038), pulmonary arterial compliance (PAC; p = 0.005), and LV filling pressures (PAWP, p = 0.039).

**Table 6:** Baseline hemodynamic characteristics of HFpEF patients, stratified by tricuspid regurgitation severity.

	All (n = 175)	Non-significant TR (48.8%)	Significant TR (51.2%)	p-value
**Hemodynamic parameters**				
**TR independent**				
sPAP (mmHg)	52.7±17.0	48.6±14.4	55.7±17.9	**0.021**
dPAP (mmHg)	22.1±7.4	20.3±6.8	23.5±7.3	**0.007**
mPAP (mmHg)	33.8±9.9	31.4±8.9	35.7±9.9	**0.009**
DPG (mmHg)	2.3±5.4	1.3±4.6	3.0±5.8	0.132
TPG (mmHg)	14.1±7.0	12.5±6.0	15.3±7.3	**0.036**
PPP (mmHg)	30.5±12.4	28.2±10.9	32.2±13.3	**0.057**
PAWP (mmHg)	19.9±5.2	19.0±5.6	20.6±4.6	**0.039**
PVR (dyn·s·cm^−5^)	226.3±141.9	188.6±93.9	257.4±168.8	**0.038**
PAC (ml/mmHg)	2.8±1.5	3.2±1.8	2.5±1.2	**0.005**
Cardiac Output (l/min)	5.3±1.3	5.5±1.4	5.1±1.2	0.085
**TR dependent**				
RAP (mmHg)	12.8±5.8	11.1±5.7	14.2±5.4	**<0.001**

TR indicates tricuspid regurgitation; sPAP, systolic pulmonary artery pressure; dPAP, diastolic pulmonary artery pressure; mPAP, mean pulmonary artery pressure; DPG, diastolic pulmonary vascular pressure gradient; TPG, transpulmonary pressure gradient; PPP, pulmonary pulse pressure; PAWP, pulmonary artery wedge pressure; PVR, pulmonary vascular resistance; PAC, pulmonary arterial compliance; RAP, right atrial pressure.

### 3.2 Factors determining the occurrence of tricuspid regurgitation

Table 7 summarizes the results of the uni- and multivariable binary logistic regression analyses. With respect to clinical parameters, atrial fibrillation was found to be independently associated with TR (p<0.001, Table 5). We also tested the association of TR- independent parameters (RV afterload) and the occurrence of TR. Multivariable analysis of hemodynamic parameters revealed diastolic PAP (p = 0.029) and PAC (p = 0.048) as independently associated with TR occurrence. Among imaging variables LA size (p = 0.001) was independently associated with the presence of significant TR.

**Table 7:** Uni- and multivariable binary logistic regression analysis for the presence of significant tricuspid regurgitation.

	B	p-value	HR (95% CI)		p-value	HR (95% CI)
UNIVARIABLE					MULITVARIABLE	
**Clinical parameters**						
Male sex	0.097	0.770	1.101	(0.576–2.106)		
Age	0.041	**0.027**	1.042	(1.005–1.081)		
BMI	-0.035	0.174	0.965	(0.917–1.016)		
BSA	-0.185	0.795	0.831	(0.207–3.341)		
Obesity	-0.258	0.482	0.773	(0.376–1.586)		
**AF**	1.635	**<0.001**	5.127	(2.620–10.034)	**<0.001**	4.864 (2.470–9.580)
Diabetes	0.003	0.991	1.003	(0.542–1.858)		
Hypertension	0.060	0.953	1.062	(0.146–7.715)		
COPD	-0.277	0.423	0.758	(0.385–1.493)		
CAD	-0.435	0.255	0.647	(0.306–1.370)		
**Imaging parameters**						
**LA diameter**	0.075	**0.001**	1.067	(1.0326–1.109)	**0.001**	1.067 (1.026–1.109)
LVEDD	-0.007	0.781	0.993	(0.942–1.046)		
IVS	-0.107	0.128	0.898	(0.782–1.031)		
LVEF	-0.003	0.681	0.997	(0.980–1.013)		
**Hemodynamic parameters**						
sPAP	0.028	**0.010**	1.028	(1.007–1.050)		
**dPAP**	0.069	**0.008**	1.071	(1.018–1.127)	**0.029**	1.061 (1.006–1.119)
mPAP	0.049	**0.008**	1.050	(1.013–1.089)		
PAWP	0.062	0.058	1.064	(0.998–1.134)		
CO	-0.245	0.054	0.783	(0.610–1.005)		
DPG	0.067	0.052	1.070	(0.999–1.145)		
TPG	0.066	**0.014**	1.068	(1.013–1.125)		
PVR	0.005	**0.004**	1.005	(1.002–1.008)		
PPP	0.028	**0.046**	1.028	(1.001–1.057)		
**PAC**	-0.321	**0.011**	0.725	(0.565–0.930)	**0.048**	0.776 (0.603–0.997)

All tested variables were determined at baseline.

TR-dependent variables were excluded from this analysis.B indicates regression correlation coefficient; HR, hazard ratio; CI, confidence interval; BMI, body mass index; BSA, body surface area; AF, atrial fibrillation; COPD, chronic obstructive pulmonary disease; CAD, coronary artery disease; LA, left atrium; LVEDD, left ventricular end-diastolic diameter; IVS, interventricular septum; LVEF, left ventricular ejection fraction; sPAP, systolic pulmonary artery pressure; dPAP, diastolic pulmonary artery pressure; mPAP, mean pulmonary artery pressure; PAWP, pulmonary artery wedge pressure; CO, cardiac output; DPG, diastolic pulmonary vascular pressure gradient; TPG, transpulmonary pressure gradient; PVR, pulmonary vascular resistance; PPP, pulmonary pulse pressure; PAC, pulmonary artery compliance.

### 3.3. Tricuspid regurgitation and event-free survival

Table 8 shows the results of the uni- and multivariable model with respect to event-free survival. Mean follow-up was 18.1±14.1 months (range: 0.1–48.0 months). Within the follow-up period, none of the patients with non-relevant TR developed relevant TR and vice-versa.

**Table 8:** Uni- and multivariable Cox regression analysis for event-free survival.

	p-value	HR (95% CI)	p- value	HR (95% CI)
UNIVARIABLE			MULTIVARIABLE	
**Clinical parameters**				
Age	0.065	1.031 (0.998–1.065)		
Male gender	0.111	1.554 (0.904–2.672)		
BMI	0.064	1.034 (0.998–1.072)		
**6-MWD**	**<0.001**	**0.995 (0.992–0.997)**	**<0.001**	**0.995 (0.992–0.998)**
AF	0.012	2.131 (1.178–3.853)		
Diabetes	0.003	2.234 (1.314–3.797)		
Hyperlipidemia	0.503	0.836 (0.495–1.412)		
Hypertension	0.506	1.957 (0.271–14.150)		
CAD	0.810	1.081 (0.571–2.047)		
**COPD**	**0.030**	**1.901 (1.064–3.397)**	**0.010**	**2.207 (1.209–4.030)**
Pacemaker	0.045	2.076 (1.016–4.245)		
NT-proBNP *	**<0.001**	**1.937 (1.478–2.539)**	**<0.001**	**1.744 (1.283–2.371)**
GFR	0.001	0.976 (0.963–0.989)		
**Hemodynamic parameters**				
**sPAP**	**<0.001**	**1.029 (1.016–1.042)**	**<0.001**	**1.029 (1.016–1.043)**
dPAP	<0.001	1.065 (1.031–1.101)		
mPAP	<0.001	1.049 (1.024–1.075)		
RAP	<0.001	1.080 (1.032–1.130)		
PAWP	0.007	1.070 (1.018–1.124)		
Cardiac output	0.753	0.967 (0.783–1.194)		
Stroke volume	0.242	1.007 (0.995–1.019)		
DPG	0.008	1.077 (1.019–1.138)		
TPG	<0.001	1.074 (1.035–1.114)		
PVR	<0.001	1.003 (1.002–1.005)		
PPP	<0.001	1.035 (1.017–1.053)		
PAC	0.016	0.720 (0.552–0.940)		
**Echocardiographic parameters**				
LVEDD	0.404	1.019 (0.974–1.067)		
LVEF	0.169	1.020 (0.992–1.049)		
LA diameter	0.008	1.043 (1.011–1.076)		
IVS	0.143	0.900 (0.781–1.036)		
RVEDD	0.001	1.058 (1.023–1.094)		
**RV FAC**	**0.009**	**0.972 (0.952–0.993)**	**0.011**	**0.9967 (0.942–0.992)**
RA diameter	0.061	1.025 (0.999–1.053)		
**E/A**	**0.006**	**1.109 (1.030–1.194)**	**0.003**	**1.147 (1.046–1.256)**
E/E’	0.104	1.039 (0.992–1.089)		
TAPSE	0.060	0.941 (0.883–1.003)		
Significant TR	0.005	2.242 (1.279–3.929)		
**Cardiac magnetic resonance imaging parameter**				
LVEDD	0.688	0.988 (0.932–1.048)		
RVEDD	0.066	1.037 (0.998–1.078)		
IVS	0.987	1.001 (0.865–1.159)		
LA	0.024	1.042 (1.005–1.080)		
RA	0.166	1.026 (0.990–1.063)		
LVEF	0.406	1.013 (0.982–1.046)		
LVEDV	0.875	0.999 (0.992–1.007)		
CO	0.643	0.954 (0.780–1.166)		
**RVEF**	**0.039**	**2.096 (1.036–4.241)**	**0.043**	**2.071 (1.024–4.189)**
RVEDV	0.802	1.000 (0.998–1.003)		

All variables were determined at baseline. Patients were followed for a mean of 18.1±14.1 months.

HR, hazard ratio; CI, confidence interval; 6-MWD, six minute walking distance; AF, atrial fibrillation; CAD, coronary artery disease; COPD, chronic obstructive pulmonary disease; GFR, glomerular filtration rate; sPAP, systolic pulmonary artery pressure; dPAP, diastolic pulmonary artery pressure; mPAP, mean pulmonary artery pressure; RAP, right atrial pressure; PAWP, pulmonary artery wedge pressure; DPG, diastolic pressure gradient; TPG, transpulmonary pressure gradient; PVR, pulmonary vascular resistance; PPP, pulmonary pulse pressure; PAC, pulmonary artery compliance; LVEDD, left ventricular end-diastolic diameter; LVEF, left ventricular ejection fraction; LA, left atrium; IVS, interventricular septal thickness; RVEDD, right ventricular end-diastolic diameter; RV FAC, right ventricular fraction area change; RA, right atrium; E/A, ratio of early to late ventricular filling velocities; E/E’, ratio of transmitral early peak velocity to septal mitral annulus velocity; TAPSE, tricuspid annular plane systolic excursion; TR, tricuspid regurgitation; LVEDV, left ventricular end-diastolic volume; CO, cardiac output; RVEF, right ventricular ejection fraction; RVEDV, right ventricular end-diastolic volume.

* NT-proBNP was analyzed by quartiles.

While TR was associated with outcome in the univariable analysis, it failed to predict event-free survival in the multivariable model. Independent predictors of cardiac events or death included 6-minute walk distance (p<0.001), chronic obstructive pulmonary disease (p = 0.010), NT-proBNP (p<0.001), E/A ratio (p = 0.003), RV dysfunction (p = 0.011) and systolic PAP (p<0.001).

## 4. Discussion

We suggest that “isolated” functional TR is a feature of HFpEF. In fact, we demonstrate here that the evolution of TR is associated with only subtle hemodynamic changes, such as reduced PAC and elevated PAP in the presence of elevated LV filling pressures. Furthermore, we show that the presence of significant TR indicates adverse outcome but is not independently associated with event-free survival.

Significant TR is a common finding [19], and has primarily been studied in patients with heart failure and reduced ejection fraction [13, 20–22] as well as those with mitral and aortic valve disease [23–25]. Thus, TR is mostly functional in nature and is thought to be the consequence of geometric alterations caused by RV dilatation, distortion of the subvalvular apparatus, tricuspid annular dilatation or a combination of these factors [26]. However, pathomechanisms underlying isolated functional TR in the absence of overt left heart pathology have not been studied.

### 4.1 Prevalence of tricuspid regurgitation in heart failure with preserved ejection fraction

We and others [27] have observed that a substantial number of consecutively enrolled HFpEF patients also suffer from relevant TR.

Despite well-established diagnostic criteria for HFpEF [2, 28] the awareness among physicians is still limited and the condition is by far under-diagnosed [29]. In a substantial number of patients presenting with shortness of breath and preserved LV systolic function, significant TR may be the only overt pathology detected by transthoracic echocardiography beneath subtle signs of diastolic dysfunction.

### 4.2 Etiology of tricuspid regurgitation in heart failure with preserved ejection fraction

In a recent publication [27], potential mechanisms underlying TR evolution in HFpEF have been discussed, attributing a role to annular dilatation due to atrial enlargement in atrial fibrillation, presence of pulmonary hypertension, or pacemaker lead impingement on the tricuspid valve leaflets. In the present study, there was no difference in TR severity between pacemaker carriers and the remainder of the group. While displacement of the right annulus in patients with atrial fibrillation is a possible mechanism of TR, the present study for the first time provides clear evidence for the pathomechanistic impact of the pulmonary circulation for TR development. In fact, pulmonary hypertension was present in both groups with and without relevant TR. However, the degree of pulmonary hypertension was more pronounced in patients with significant TR, reflected by slightly higher pulmonary pressures. In the multivariable regression model diastolic PAP was identified as a parameter independently associated with relevant TR. Moreover, PAC was lower in the TR group compared with the non-TR group and also remained independently associated with relevant TR.

PAC in post-capillary pulmonary hypertension is dependent on PAWP [30]. Indeed, PAWP was significantly higher in the TR versus non-TR group.

### 4.3 Significance of tricuspid regurgitation in heart failure with preserved ejection fraction

With respect to event-free survival, relevant TR failed to predict outcome in the Cox regression analysis (Table 8). This is in line with a recent report by Mohammed et al [27] where RV dysfunction but not TR was an independent predictor of adverse outcome. These findings suggest that the presence of functional relevant TR is a bystander or marker of disease, but not a stand-alone pathology in HFpEF.

### 4.4 Right ventricular dysfunction in heart failure with preserved ejection fraction

**Fig 1:**
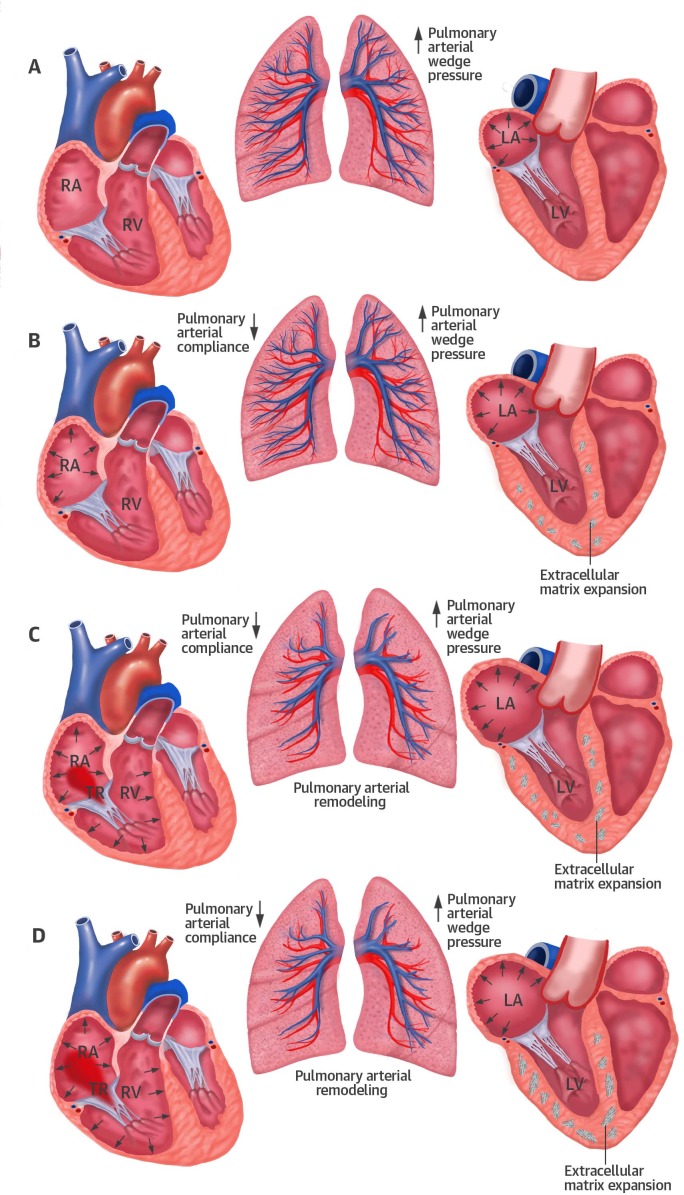
Pathomechanistic processes underlying the development of tricuspid regurgitation in heart failure with preserved ejection fraction.

In contrast to non-TR patients, those with significant TR had larger right heart dimensions and worse RV function by echo as well as CMR studies. RV dysfunction was an independent predictor of event-free survival in the present study (Table 8), confirming previous publications [6, 8, 27]. As illustrated in Fig 1, RV dysfunction ensues elevation of LV filling pressures causing a passive and—occasionally–also active rise in PAP due to pulmonary vascular remodeling [31]. As soon as significant TR develops due to RV dilatation, volume overload adds to the pre-existing pressure overload, thereby promoting the vicious circle of RV failure. In the presence of significant TR, the degree of RV systolic dysfunction may frequently be underestimated in analogy to LV systolic function in the presence of mitral regurgitation. Because RV dysfunction is a key determinant of prognosis, more emphasis should be put on its evaluation, in particular in the presence of relevant TR.

HFpEF is characterized by impaired left ventricular (LV) diastolic function due to abnormal relaxation and increased chamber stiffness. The consecutive rise in LV filling pressure results in a passive rise of pulmonary arterial pressure (PAP, Panel A). As a consequence, pulmonary vascular compliance declines and adds to the increasing resistance against the right ventricle (RV, Panel B). Remodeling of the pre-capillary pulmonary vascular bed may occur as an additional mechanism aggravating RV pressure overload (Panel C). The RV fails to compensate pressure overload and dilates. Tricuspid annular dilatation and distortion of the subvalvular apparatus lead to increasing tricuspid regurgitation and consecutive right heart failure (Panel D).

Reprinted with permission from John Wiley and Sons, from Aschauer et al. [8], license number 3858290307001.

## 5. Limitations

The present study has been undertaken in a single center with a relatively small sample size. A center-specific bias cannot be excluded. However, the major advantages of limiting data collection to a single center are 1. inclusion of a homogenous patient population, 2. constant clinical routine, 3. constant quality of assessment techniques and 4. constant follow-up.

CO derived by the thermodilution method may be confounded by the presence of relevant TR. However, there was a tight correlation between this method and additional CO measurements, including the Fick method (r = 0.631, p<0.001) and the CMR-derived CO (r = 0.515, p<0.001). Parameters of LA function have not been assessed in the present study.

## 6. Conclusions

The diagnosis of ´isolated´ functional TR should prompt further evaluation of the LV, in particular with respect to the presence of LV diastolic dysfunction. In addition to non-invasive assessment, right heart catheter to determine the relation between pulmonary pressures and flow should be considered, since only subtle changes may be associated with relevant TR. Although patients with significant TR face a dismal prognosis, TR itself is not independently associated with outcome. Our data suggest that isolated TR is a bystander of HFpEF and the necessity of therapeutic interventions, such as tricuspid valve surgery, should be questioned.
